# Pressure-induced zigzag phosphorus chain and superconductivity in boron monophosphide

**DOI:** 10.1038/srep08761

**Published:** 2015-03-04

**Authors:** Xinyu Zhang, Jiaqian Qin, Hanyu Liu, Shiliang Zhang, Mingzhen Ma, Wei Luo, Riping Liu, Rajeev Ahuja

**Affiliations:** 1 State Key Laboratory of Metastable Materials Science and Technology, Yanshan University, Qinhuangdao 066004, P.R. China; 2 Metallurgy and Materials Science Research Institute, Chulalongkorn University, Bangkok 10330, Thailand; 3 Department of Physics and Engineering Physics, University of Saskatchewan, Saskatchewan, Canada, S7N 5E2; 4 Condensed Matter Theory Group, Department of Physics and Astronomy, Uppsala University, SE-751 20 Uppsala, Sweden; 5 Applied Material Physics, Department of Materials Science and Engineering, Royal Institute of Technology, SE-100 44 Stockholm, Sweden; 6 Quantum Functional Semiconductor Research Center, Physics Department, Dongguk University, Chung gu, Seoul 100-715, Korea

## Abstract

We report on the prediction of the zinc-blende structure BP into a novel *C*2/*m* phase from 113 to 208 GPa which possesses zigzag phosphorus chain structure, followed by another *P*4_2_/*mnm* structure above 208 GPa above using the particle-swarm search method. Strong electron-phonon coupling λ in compressed BP is found, in particular for *C*2/*m* phase with the zigzag phosphorus chain, which has the highest λ (0.56–0.61) value among them, leading to its high superconducting critical temperature *T_c_* (9.4 K–11.5 K), which is comparable with the 4.5 K to 13 K value of black phosphorus phase I (orthorhombic, *Cmca*). This is the first system in the boron phosphides which shows superconductivity from the present theoretical calculations. Our results show that pressure-induced zigzag phosphorus chain in BP exhibit higher superconducting temperature *T_C_*, opening a new route to search and design new superconductor materials with zigzag phosphorus chains.

The crucial thermodynamic parameter-high pressure has been emerging as a powerful tool to investigate physical and chemical behaviours of materials, especially to synthesize or design materials with excellent properties such as superhardness and superconductivity^1^,^2^,^3^,^4^,^5^,^6^. Recently, B and P have been studied extensively in physical, chemical, and material science fields due to their interesting structural properties when pressure is applied^4^,^5^,^7^,^8^,^9^. Boron has always been recognized as a complex element, both structurally and electronically: its crystalline phases are numerous and inevitably complicated, which is related to its electron deficiency and thus the tendency to form multicenter bonds^5^,^7^. Phosphorus, on the other hand, has six known phases at room temperature under high pressure^10^,^11^,^12^,^13^,^14^,^15^. Black phosphorus phase I (orthorhombic, *Cmca*) is known to be most stable under ambient temperature and pressure^15^. The experiment at low temperature and high pressure showed that the superconducting temperature of phase I increased from 4.5 K to 13 K under pressure^14^. These peculiar physical properties of compressed B and P solids have motivated our attention on high-pressure study of BP. The binary semiconductor boron phosphide (BP) is the zinc-blende crystal structure (space group *F*-43*m*) with lattice parameter a = 4.537 Å^16^, which has extraordinary properties, such as high hardness, high temperature stability, resistance to chemical corrosion, high thermoelectric powers for direct energy conversion, and is regarded as an important candidate for electronic, optical, and other engineering applications^17^,^18^,^19^,^20^,^21^. In order to understand in detail the pressure-induced structural behaviour, including mainly the phase transitions of BP, further high-pressure studies are essential.

Here, on the basis of comprehensive density functional theory (DFT) computations, we report the design of two new high pressure structures of BP. We performed variable-cell structure prediction simulations using CALYPSO^22^,^23^ approach for BP at 0, 50, 100, 150, 200, 250, and 300 GPa, respectively. For comparison, the structures reported in boron nitride (*P-3m1*, *P6_3_mc*, *P6_3_/mmc*) experimentally and theoretically, are also considered in our calculations. Our calculations demonstrate that, *F*-43*m* is the most stable phase at ambient conditions, two new high pressure phases *C*2/*m* and *P*4_2_/*mnm* can be predicted during compression up to 300 GPa. We further elucidate the energetic, mechanical, electronic and superconducting properties of the obtained novel phases, confirming that *F*-43*m* is semiconductor, and discovering two high-pressure superconducting phases *C*2/*m* and *P*4_2_/*mnm*.

## Results

**Figure 1:**
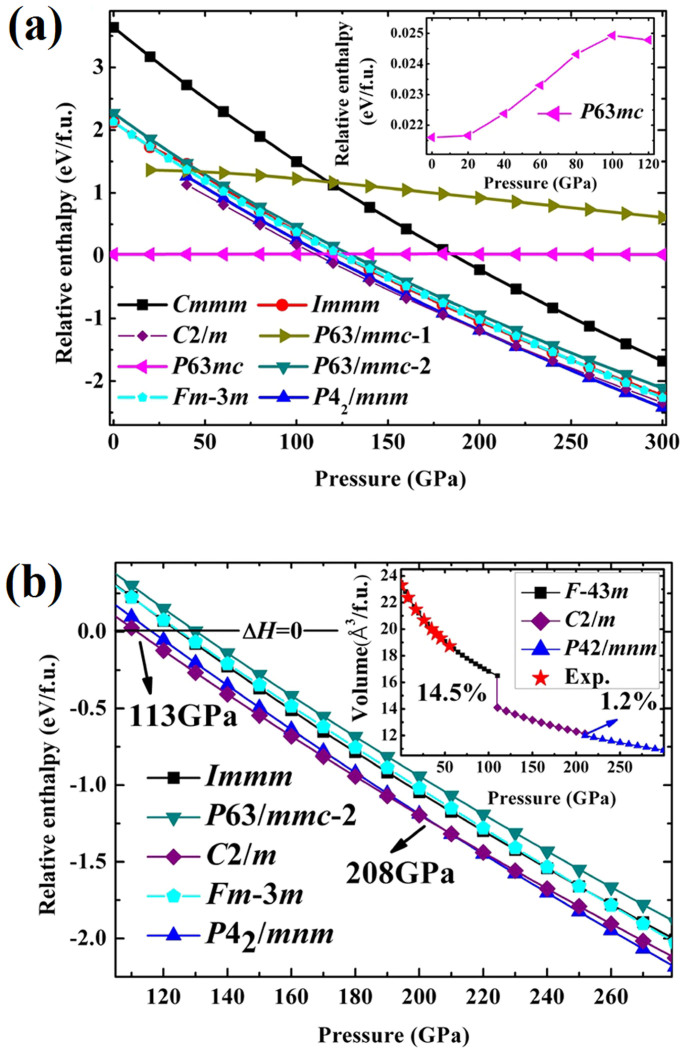
(a) The relative enthalpy per formula unit as a function of pressure for competing structures. The enthalpies are referenced to that of *F*-43*m* phase. The insert is the enlarged view of relative enthalpy of *P*63*mc* phase as a function of pressure. (b) The relative enthalpy per f.u. as a function of pressure with the region from 105 GPa to 280 GPa around the phase transition pressure. The insert is the pressure dependence of volume for *F*-43*m*, *C*2/*m* and *P*42/*mnm* phases of BP.

The analysis of the predicted structures gives us a list of candidate structures with space groups *F*-43*m, C*2/*m, P*4_2_/*mnm, P*6_3_*mc, Cmmm, Immm, P63/mmc-I*, *P63/mmc-II*, and the previously reported high pressure rock salt structure *Fm-3m*^20^ are depicted in Figure S1 (Supporting Information). The relative enthalpies per chemical formula unit vs. pressure curves of selected structures are plotted in Figure 1(a). Considering that *F-43m* and *P*6_3_*mc* structures hold very close enthalpy, the enthalpy difference vs. pressure curves for the two phases are specially inserted in Figure 1(a), and it indicates that *F-43m* has lower enthalpy, which is consistent with the experimental results. In order to investigate the phase transition pressures clearly, the enthalpies vs. pressure curves for *F-43m*, *Immm, P*4_2_/*mnm, C*2/*m,* and *P*-3*m*1 are given in Figure 1(b). From Figure 1, one can see that, for compressed BP, the zinc-blende crystal structure *F-43m* is the most stable structure below 113 GPa, a *C*2/*m* phase takes over the pressure range from 113 to 208 GPa, followed by another *P*4_2_/*mnm* structure above 208 GPa. The pressure evolution of the unit cell volume of BP in the structures *F*-43*m*, *C*2/*m*, and *P*4_2_/*mnm* is inserted in Figure 1(b). The experimental equation of state data^24^ for single-crystal BP with *F-43m* structure up to 55 GPa were listed for comparison. It can be seen that our calculated data are in good agreement with the experimental results. The zinc-blende crystal structure *F-43m* is stable on compression to 113 GPa, and then complete change to *C*2/*m* is observed. Comparisons with the *F-*43*m* cell at 113 GPa shows a 14.5% reduction in volume. Upon further compression into the region of *P*4_2_/*mnm* above 208 GPa, the volume difference peaks at only 1.2%.

**Figure 2:**
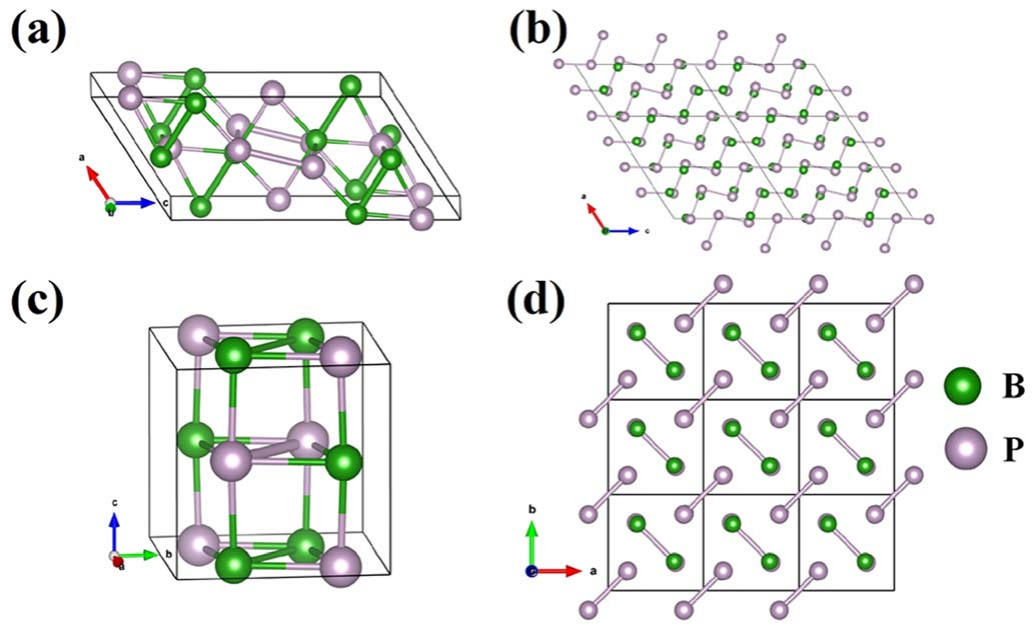
Predicted structures of BP at ambient and high pressures. The green and pink balls represent B and P atoms, respectively. (a) and (b) The high-pressure phase *C*2/*m*. (c) and (d) The high-pressure phase *P*42/*mnm*. One-dimensional phosphorus chain is formed in the structure of *C*2/*m*.

The optimized structural parameters of zinc-blende crystal structure *F-43m* are listed in Table S1. For this phase, the equilibrium lattice constants are 4.547 Å and 4.492 Å, for PBE and LDA calculation, respectively. The present results are in good agreement with the previous results^21^,^25^,^26^,^27^. In this structure, four boron atoms lie in the Wyckoff 4*a* site and four phosphorus atoms occupy the 4*c* site, in which three-dimensional (3D) boron and phosphorus network in this structure is formed, as shown in Figure S1. The optimized structural parameters of another two phases *C*2/*m* and *P*4_2_/*mnm* at high pressure are presented in Table 1. For monoclinic *C*2/*m* phase, the equilibrium lattice constants at 120 GPa are *a* = 4.275 Å, *b* = 3.681 Å, *c* = 8.357 Å and *β* = 122.627 degree. The structure of *C*2/*m* is shown in Figure 2(a) and Figure 2(b), it can be seen that the 1D-zigzag phosphorus chain is formed, which is connected by boron atoms. For hexagonal *P*4_2_/*mnm* phase (Figure 2(c)), the equilibrium lattice constants at 210 GPa are *a* = 3.631 Å, *c* = 3.640 Å. The B-P, B-B, and P-P bonds are formed in this structure, but P-P bonds are not continuous (Figure 2(d)).

**Figure 3:**
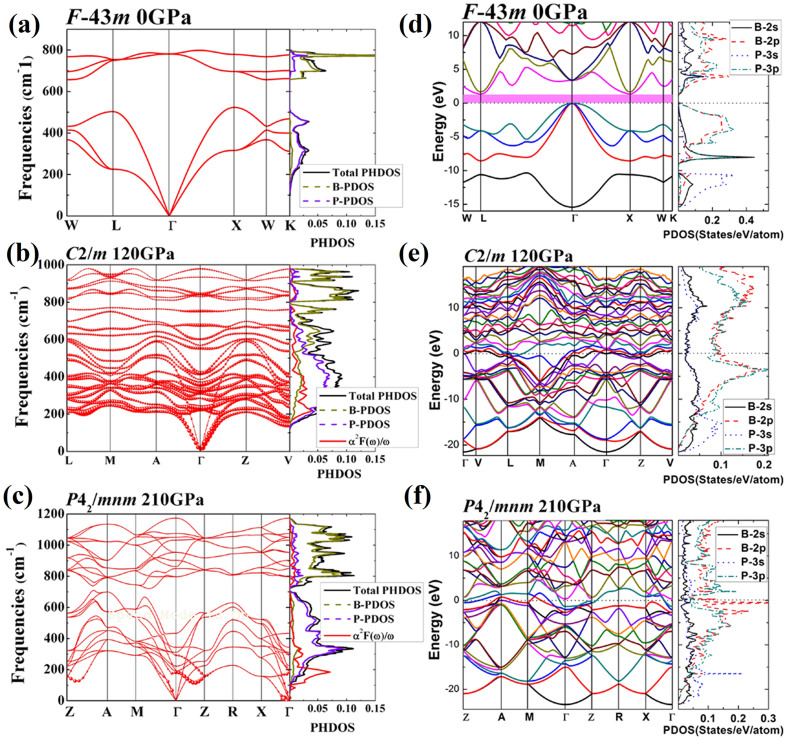
Phonon spectrum and electronic structures. (a) The phonon spectrum and partial atomic phonon density of states for phase *F*-43*m*. (b) The phonon spectrum, partial atomic phonon density of states and Eliashberg phonon spectral function α^2^*F*(ω)/ω for phase *C*2/*m*. (c) The phonon spectrum, partial atomic phonon density of states and Eliashberg phonon spectral function α^2^*F*(ω)/ω for phase *P*42/*mnm*. Red solid circles in (b) and (c) show the electron-phonon coupling with the radius proportional to their respective strength. Energy band and projected density of states (PDOS) for phase (d) *F*-43*m*, (e) *C*2/*m*, and (f)*P*42/*mnm*.

The calculated elastic constants for three BP phases with low enthalpy are presented in Table 2. From Table 2, the elastic constants reveal that all BP phases satisfy the mechanical stable criterion at the corresponding pressures. Furthermore, the hexagonal phase *P*4_2_/*mnm* can remain stable at ambient pressure, but monoclinic *C*2/*m* does not match the mechanical stable criterion at ambient pressure, which only keeps stable at the high pressure range. Also, the calculated negative formation enthalpies of *C*2/*m* and *P*4_2_/*mnm* phases at the pressure ranges indicate the stability against decomposition of BP into the elements boron and phosphorus^7^. Phonon calculations show that BP phases (*F-43m*, *C*2/*m* and *P*4_2_/*mnm*) are dynamically stable at their thermodynamically stable pressure regions. Phonon dispersions and partial phonon density of states (PPHDOS) of BP phases at their stable pressure ranges are shown in Figure 3. The maximum optical branch frequencies are 803.5 cm^−1^ for *F-43m* phase at atmospheric pressure, 983.7 cm^−1^ for *C*2/*m* phase at 120 GPa, and 1175.2 cm^−1^ for *P*4_2_/*mnm* phase at 210 GPa. One can see that the maximum optical branch frequencies increase with increasing pressure due to an obvious structural difference. From PPHDOS, the lower frequency region is associated with phosphorus atoms, whereas boron atom contributes to the high-frequency region for high-pressure phases (*C*2/*m* and *P*4_2_/*mnm*) at their corresponding pressure regions. But in the *F-43m* phase, the boron atom dominates the high-frequency region, whereas phosphorus atom contributes almost equally to the high and low frequency region.

**Figure 4:**
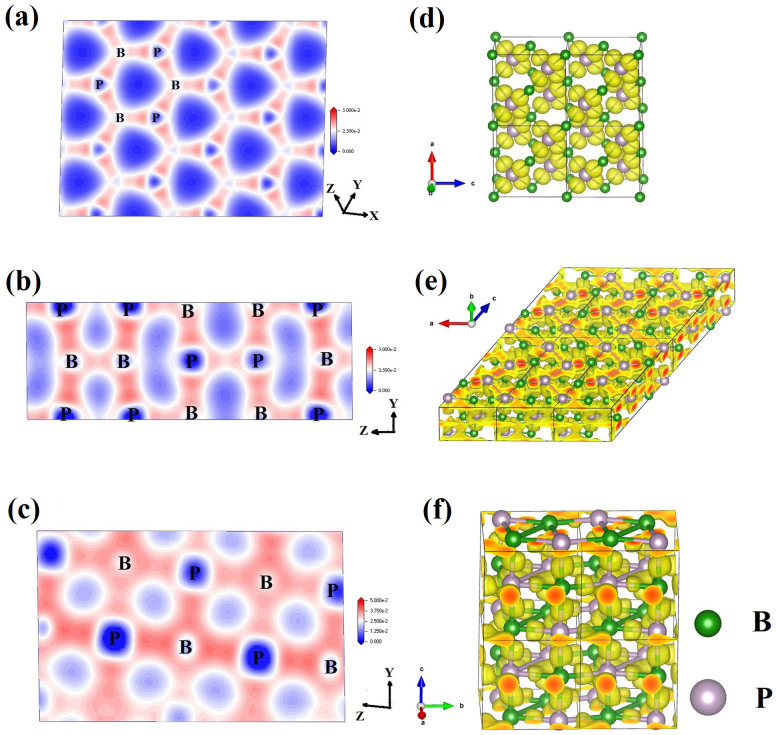
Electron density and Electron localization function(ELF). (a), (b), and (c) Electron density for phase *F*-43*m*, *C*2/*m*, and *P*42/*mnm*, respectively. (d) Calculated ELF with an isosurface ELF value of 0.8, 0.65, and 0.7 for phase *F*-43*m*, *C*2/*m*, and *P*42/*mnm*, respectively.

To investigate the possibility of pressure induced metallization and superconducting transition, band structures for *F-43m*, *C*2/*m* and *P*4_2_/*mnm* phases have been calculated to examine the electronic properties of these polymorphs of BP at their pressure ranges as shown in Figure 3. Electronic structure calculations indicate that *F-43m* phase is a semiconductor, whereas *C*2/*m* and *P*4_2_/*mnm* phases are metallic. From projected density of states (PDOS) plotted in Figure 3, it can be observed that all phases of BP have consistent electronic distributions. B-2*p* and P-3*p* electrons dominate in the wide energy range and form the strong covalent bonds as suggested by the matching B-2*p* and P-3*p* curve shapes. The contour plots of charge density on the chosen planes are also shown in Figure 4. The strong covalent B-P bonding within in the 3D B-P network is evidenced (Figures 4(a), 4(b) and 4(c)). Moreover, according to Mulliken population analysis, P-P bonding does not exist in *F-43m* phase, but it exists in *C*2/*m* and *P*4_2_/*mnm* phases. Interestingly, P-P zigzag chains in phase *C*2/*m* transfer to discontinuous bonding in phase *P*4_2_/*mnm*. This conclusion is further substantiated by the electron localization function (ELF) shown in Figures 4(d), 4(e) and 4(f), where the calculated ELF patterns have isosurface values of 0.8, 0.65, and 0.7 for *F-43m*, *C*2/*m*, and *P*4_2_/*mnm*, respectively.

**Figure 5:**
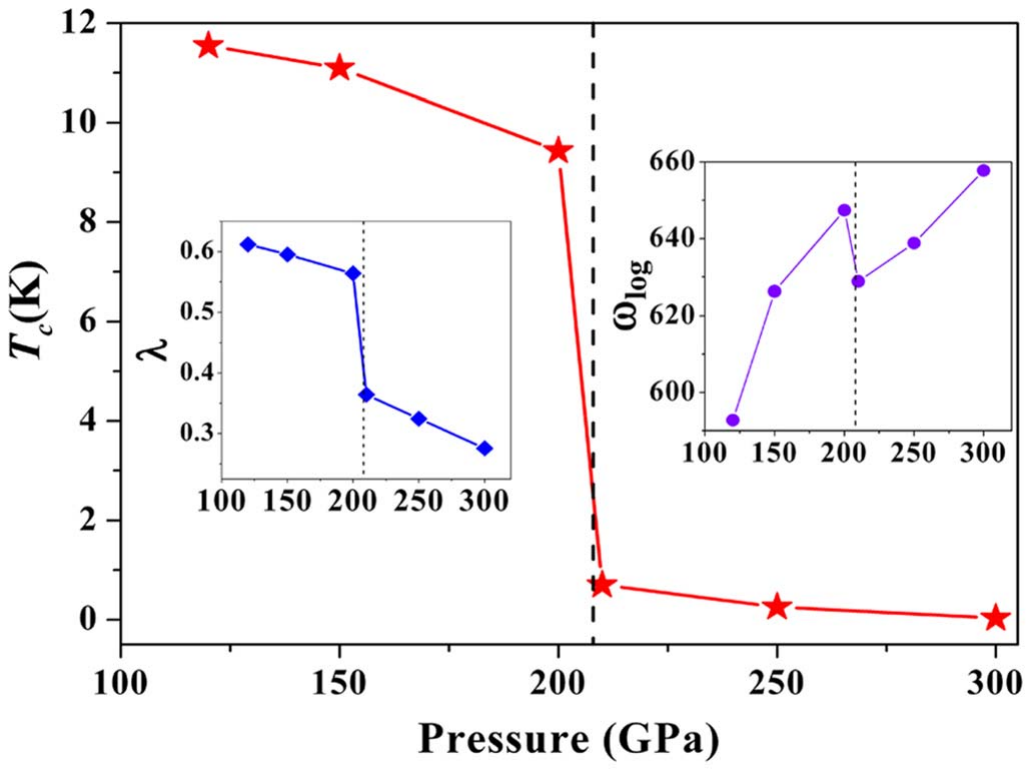
Superconducting temperature (TC) vs. pressure. The coupling parameters, the average phonon frequencies ω_ln_ and superconducting temperatures of BP as a function of pressure.

The high-pressure *C*2/*m* phase of BP exhibits interesting features in its electronic band structure. As shown in Figure 3(e), the electronic bands of the *C*2/*m* phase crossing the Fermi level along the Γ -V-L and A-Γ-Z-V are quite flat, and this section of band within a narrow energy window centered at the Fermi level gives rise to large electronic density of states near the Fermi energy. The corresponding confined conduction electrons near the band gap possess large effective mass with their group velocities approaching zero. In contrast, the bands along the L-M-A direction steeply cross the Fermi level, providing itinerant electrons with high conduction electron velocity. Such flat bands with highly mobile and localized electrons have been shown to enhance superconductivity^28^,^29^,^30^. Similar electronic structures have previously been found in niobium that has the highest transition temperature *T_C_* of 9.3 K^31^ in all element superconductors at ambient pressure, 9.8 K in CaC_2_^28^ and 220–235 K in CaH_6_ at high pressure of 150 GPa^32^. This motivates us to further investigate the superconductivity of BP at high pressures. The calculated spectral function α^2^*F*(ω) of BP phases *C*2/*m* and *P*4_2_/*mnm* at 120 GPa and 210 GPa were plotted in Figures 3(b) and 3(c). At 120 GPa (Figure 3(b)), the coupling parameter λ is 0.61 with the average phonon frequency ω_ln_ of 593 K. Using the strong coupling Allen-Dynes equation, an extension of the McMillan theory, with a nominal Coulomb pseudopotential parameter (μ*) of 0.12 the estimated superconducting critical temperature *T_C_* is 11.5 K. With increasing pressure, the calculated *T_C_* become slightly lower as 11.1 K at 150 GPa and 9.4 K at 200 GPa due to slightly smaller λ of 0.59 at 150 GPa and 0.56 at 200 GPa, respectively. Here the *T_C_* of two high-pressure phases for BP and *T_C_*'s dependence on pressure is shown in Figure 5. Calculated coupling parameter λ values and logarithmic phonon momentum ω_log_ vs. pressure curves are also presented in Figure 5. It can be seen that logarithmic phonon momentum ω_log_ increases with pressure, and coupling parameter λ decreases with pressure. Among these two high pressure phases, *C*2/*m* phase has the strongest electron-phonon coupling and so has the highest *T_C_*. Apparently, the fairly high *T_C_* is due to the large electron phonon coupling (λ). The origin can be traced from comparing the calculated Eliashberg spectral function (α^2^*F*(ω)/ω) with the projected phonon DOS. As shown in Figure 3(b), nearly 80% of the electron phonon coupling is contributed by the low-frequency vibrations in the frequency region from 100 to 600 cm^−1^. The low-frequency vibrations in the frequency region from 100 to 600 cm^−1^, which are mostly attributed to the P atom. At wide range of pressure, *C*2/*m* phase has comparative superconducting critical temperatures (from 11.5 K at 120 GPa to 9.3 K at 200 GPa) with 11.5 K value of CaC_6_^33^,^34^. However, *T_C_* of BP is close to zero above 210 GPa due to the smaller electron-phonon coupling parameter (λ < 0.4). We thus further analyze the coupling parameter in each q-point (Figures 3(b) and 3(c)). It is clearly seen that the coupling parameters in Γ-Z and X-Γ are very strong. This superconducting feature is typical anisotropy, leading a low superconductivity value.

To summarize, using the PSO technique on crystal structure prediction, we designed two new high-pressure phases of BP, *C*2/*m* and *P*4_2_/*mnm*, which are stable in the pressure ranges of 113–208 GPa and 208–300 GPa, respectively. Elastic constants and phonon calculations have shown their mechanical and dynamical stability at the dominating pressure ranges. Our calculations reveal that the phosphorus atomic arrangement form from single atoms to 1D zigzag chains to discontinuous bonding. The high superconducting transition temperature of the *C*2/*m* phase benefits from the simultaneous presence of the steep bands (highly mobile electrons) and extremely flat bands (highly confined electrons), which is known to favor the electron paring and superconducting behavior.

## Computational Methods

The search of high-pressure structures was performed with variable-cell PSO^35^ structure prediction simulations using CALYPSO approach^22^,^23^ in combined with Vienna *ab initio* simulation package (VASP)^36^. CALYPSO was designed to predict stable or metastable crystal structures requiring only chemical compositions of a given compound at specified external conditions. All structures were locally optimized using the DFT method. The 60% structures of each generation with lower enthalpies were selected to generate the structures for the next generation by PSO operation, and the other structures in new generation were randomly generated to increase the structural diversity. The underlying ab initio calculations were performed using density functional theory within the generalized gradient approximation (GGA)^37^, as implemented in the Vienna *ab initio* simulation package (VASP). The total energy calculations were carried out with the VASP code. The all-electron projector augmented wave (PAW) method^38^ was employed with a plane-wave cutoff energy of 600 eV for all phases. The k-point samplings in the Brillouin zone were performed using the Monkhorst-Pack scheme^39^. The total energy convergence tests showed that convergence to within 1 meV/atom was achieved with the above calculation parameters. Single crystal elastic constants were calculated via a strain-stress approach. i.e., by applying a small strain to the equilibrium lattice of the unit cell and fitting the dependence of the resulting stress on the strain. The bulk modulus, shear modulus, Young's modulus, and Poisson's ratio were determined by using the Voigt-Reuss-Hill approximation^40^. The lattice-dynamical and superconducting properties are calculated using the density functional perturbation theory (DFPT)^41^ as implemented in the Quantum Espresso package^42^ using with the Troullier-Martins pseudopotentials with cutoff energies of 50 and 500 Ry for the wave functions and the charge density, respectively. Fine k-points mesh of MP was used and the estimated energy error in self-consistency was less than 10^−4^ a.u. The electron–phonon coupling was convergent with a finer grid and a Gaussian smearing of 0.02 Ry. In order to check the results, the phonon calculations were also carried out by using a supercell approach as implemented in the PHONOPY code^43^.

## Author Contributions

X.Z., J.Q., R.L. and R.A. designed and coordinated the research. X.Z., J.Q., H.L. and S.Z. did the calculations. X.Z., J.Q., H.L., S.Z., M.M., R.L., W.L. and R.A. analyzed all data. X.Z., J.Q., H.L., R.L. and R.A. wrote the manuscript.

## Supplementary Material

Supplementary Information Supplementary information

## Figures and Tables

**Table 1:** Optimized structural parameters for two high-pressure structures of BP

Space group	Pressure (GPa)	Lattice parameters (Å or degree)	Atomic coordinates (fractional)
*C*2/*m*	120	*a* = 4.275, *b* = 3.681, *c* = 8.357	B1(4*i*)(1.48905, 0.00000, 0.89375)
			B2(4*i*)(1.05110, 0.00000, 0.65390)
		*α* = *γ* = 90, *β* = 122.627	P1(4*i*)(0.99081, 0.00000, 0.13299)
			P2(4*i*)(0.56959, 0.00000, 0.39201)
*P*4_2_/*mnm*	210	*a* = 3.631, *c* = 3.640	B (4*g*)(0.30702, 0.69298, 0.00000)
			P (4*f*) (0.70572, 0.29428, 0.50000)

**Table 2:** Independent elastic constants *C*_ij_, bulk, shear, and Young's moduli (*B*, *G* and *E* all in GPa), Poisson's ratio *ν* and *G*/*B* of the stable phases of BP

Space group	*P*	*C*_11_	*C*_22_	*C*_33_	*C*_44_	*C*_55_	*C*_66_	*C*_12_	*C*_13_	*C*_15_	*C*_23_	*C*_25_	*C*_35_	*C*_46_	*B*	*G*	*E*	*ν*	*G*/*B*
*F*-43*m*	0	340			189			73							162	165	369	0.120	1.016
*C*2/*m*	120	761	1327	830	390	268	449	313	451	−10	355	6	−35	−15	563	319	805	0.261	0.567
*P*4_2_/*mnm*	210	1326		2025	575		78	670	350						821	354	929	0.311	0.431

## References

[b1] McMillan P. F. Chemistry at high pressure. Chem. Soc. Rev. 35, 855–857 (2006). 17003892 10.1039/b610410j

[b2] Haines J., Léger J. & Bocquillon G. Synthesis and design of superhard materials. Ann. Rev. Mater. Res. 31, 1–23 (2001).

[b3] Qin J. *et al.* Is Rhenium Diboride a Superhard Material? Adv. Mater. 20, 4780–4783 (2008).

[b4] Qin J. *et al.* Polycrystalline γ-boron: As hard as polycrystalline cubic boron nitride. Scripta Mater. 67, 257–260 (2012).

[b5] Qin J. *et al.* Phase relations in boron at pressures up to 18 GPa and temperatures up to 2200°C. Phys. Rev. B 85, 014107 (2012).

[b6] Raza Z., Errea I., Oganov A. R. & Saitta A. M. Novel superconducting skutterudite-type phosphorus nitride at high pressure from first-principles calculations. Sci. Rep. 4, 5889; 10.1038/srep05889 (2014). PMC4115206 25074347

[b7] Oganov A. R. *et al.* Ionic high-pressure form of elemental boron. Nature 457, 863–867 (2009). 19182772 10.1038/nature07736

[b8] Ma Y., Prewitt C. T., Zou G., Mao H.-k. & Hemley R. J. High-pressure high-temperature x-ray diffraction of β-boron to 30 GPa. Phys. Rev. B 67, 174116 (2003).

[b9] Ma Y., Tse J. S., Klug D. D. & Ahuja R. Electron-phonon coupling of α-Ga boron. Phys. Rev. B 70, 214107 (2004).

[b10] Kikegawa T. & Iwasaki H. An X-ray diffraction study of lattice compression and phase transition of crystalline phosphorus. Acta Crystallogr. B 39, 158–164 (1983).

[b11] Akahama Y., Kobayashi M. & Kawamura H. Simple-cubic–simple-hexagonal transition in phosphorus under pressure. Phys. Rev. B 59, 8520–8525 (1999).

[b12] Akahama Y., Kawamura H., Carlson S., Le Bihan T. & Häusermann D. Structural stability and equation of state of simple-hexagonal phosphorus to 280 GPa: Phase transition at 262 GPa. Phys. Rev. B 61, 3139–3142 (2000).

[b13] Akahama Y., Endo S. & Narita S. Electrical properties of single-crystal black phosphorus under pressure. Physica B + C 139–140, 397–400 (1986).

[b14] Kawamura H., Shirotani I. & Tachikawa K. Anomalous superconductivity in black phosphorus under high pressures. Solid State Commun. 49, 879–881 (1984).

[b15] Brown A. & Rundqvist S. Refinement of the crystal structure of black phosphorus. Acta Crystallographica 19, 684–685 (1965).

[b16] Peret J. L. Preparation and Properties of the Boron Phosphides. J. Am. Ceram. Soc. 47, 44–46 (1964).

[b17] Archer R. J., Koyama R. Y., Loebner E. E. & Lucas R. C. Optical Absorption, Electroluminescence, and the Band Gap of BP. Phys. Rev. Lett. 12, 538–540 (1964).

[b18] Motojima S., Ohtsuka Y., Kawajiri S., Takahashi Y. & Sugiyama K. Boron phosphide coatings on molybdenum by chemical vapour deposition, and their composition and microhardness. J. Mater. Sci. 14, 496–498 (1979).

[b19] Schroten E., Goossens A. & Schoonman J. Photo- and electroreflectance of cubic boron phosphide. J. Appl. Phys. 83, 1660–1663 (1998).

[b20] Wentzcovitch R. M., Cohen M. L. & Lam P. K. Theoretical study of BN, BP, and BAs at high pressures. Phys. Rev. B 36, 6058–6068 (1987). 10.1103/physrevb.36.6058 9942288

[b21] Wentzcovitch R. M., Chang K. J. & Cohen M. L. Electronic and structural properties of BN and BP. Phys. Rev. B 34, 1071–1079 (1986). 10.1103/physrevb.34.1071 9939723

[b22] Wang Y., Lv J., Zhu L. & Ma Y. Crystal structure prediction via particle-swarm optimization. Phys. Rev. B 82, 094116–094123 (2010).

[b23] Wang Y., Lv J., Zhu L. & Ma Y. CALYPSO: A method for crystal structure prediction. Comput. Phys. Commun. 183, 2063–2070 (2012).

[b24] Godec Y. *et al.* Equation of state of single-crystal cubic boron phosphide. J. Superhard Mat. 36, 61–64 (2014).

[b25] Meradji H. *et al.* First-principles elastic constants and electronic structure of BP, BAs, and BSb. Phys. Status. Solidi. B 241, 2881–2885 (2004).

[b26] Lambrecht W. R. L. & Segall B. Electronic structure and bonding at SiC/AlN and SiC/BP interfaces. Phys. Rev. B 43, 7070–7085 (1991). 10.1103/physrevb.43.7070 9998171

[b27] Zaoui A. & Hassan F. E. H. Full potential linearized augmented plane wave calculations of structural and electronic properties of BN, BP, BAs and BSb. J. Phys.: Condens. Matter 13, 253–262 (2001).

[b28] Li Y.-L. *et al.* Pressure-induced superconductivity in CaC_2_. Proc. Natl. Acad. Sci. USA 110, 9289–9294 (2013). 23690580 10.1073/pnas.1307384110 PMC3677455

[b29] Ranninger J., Robin J. M. & Eschrig M. Superfluid Precursor Effects in a Model of Hybridized Bosons and Fermions. Phys. Rev. Lett. 74, 4027–4030 (1995). 10058394 10.1103/PhysRevLett.74.4027

[b30] Simon A. Superconductivity and chemistry. Angew. Chem. Int. Ed. 36, 1788–1806 (1997).

[b31] Broom R. F., Raider S. I., Oosenbrug A., Drake R. E. & Walter W. Niobium oxide-barrier tunnel junction. Ieee. T. Electron Dev. 27, 1998–2008 (1980).

[b32] Wang H., Tse J. S., Tanaka K., Iitaka T. & Ma Y. Superconductive sodalite-like clathrate calcium hydride at high pressures. Proc. Natl. Acad. Sci. USA 109, 6463–6466 (2012). 22492976 10.1073/pnas.1118168109 PMC3340045

[b33] Emery N. *et al.* Superconductivity of Bulk CaC_6_. Phys. Rev. Lett. 95, 087003 (2005). 16196893 10.1103/PhysRevLett.95.087003

[b34] Weller T. E., Ellerby M., Saxena S. S., Smith R. P. & Skipper N. T. Superconductivity in the intercalated graphite compounds C_6_Yb and C_6_Ca. Nat. Phy. 1, 39–41 (2005).

[b35] Call S. T., Zubarev D. Y. & Boldyrev A. I. Global minimum structure searches via particle swarm optimization. J. Comput. Chem. 28, 1177–1186 (2007). 17299774 10.1002/jcc.20621

[b36] Kresse G. & Furthmüller J. Efficient iterative schemes for ab initio total-energy calculations using a plane-wave basis set. Phys. Rev. B 54, 11169–11186 (1996). 10.1103/physrevb.54.11169 9984901

[b37] Perdew J. P., Burke K. & Ernzerhof M. Generalized gradient approximation made simple. Phys. Rev. Lett. 77, 3865–3868 (1996). 10062328 10.1103/PhysRevLett.77.3865

[b38] Kresse G. & Joubert D. From ultrasoft pseudopotentials to the projector augmented-wave method. Phys. Rev. B 59, 1758–1775 (1999).

[b39] Monkhorst H. J. & Pack J. D. Special points for Brillouin-zone integrations. Phys. Rev. B 13, 5188–5192 (1976).

[b40] Hill R. The Elastic Behaviour of a Crystalline Aggregate. Proc. Phys. Soc. A 65, 349–355 (1952).

[b41] Baroni S., de Gironcoli S. & Dal Corso A., Giannozzi P. Phonons and related crystal properties from density-functional perturbation theory. Rev. Mod. Phys. 73, 515–562 (2001).

[b42] Giannozzi P. *et al.* QUANTUM ESPRESSO: a modular and open-source software project for quantum simulations of materials. J. Phys.: Condens. Matter 21, 395502–395530 (2009). 21832390 10.1088/0953-8984/21/39/395502

[b43] Togo A., Oba F. & Tanaka I. First-principles calculations of the ferroelastic transition between rutile-type and CaCl_2_-type SiO_2_ at high pressures. Phys. Rev. B 78, 134106–134114 (2008).

