# Supplementary material for: Pressure-induced zigzag phosphorus chain and superconductivity in boron monophosphide
Source: Sci Rep. 2015 Mar 4;5:8761. doi: 10.1038/srep08761 (PMC4348669; doi:10.1038/srep08761)
Supplement: Supplementary Information [file srep08761-s1.doc]

**Supporting information**

Pressure-induced zigzag phosphorus chain and superconductivity in boron monophosphide

Xinyu Zhang, Jiaqian Qin,*Hanyu Liu, Shiliang Zhang, Mingzhen Ma, Wei Luo, Riping Liu,* Rajeev Ahuja*


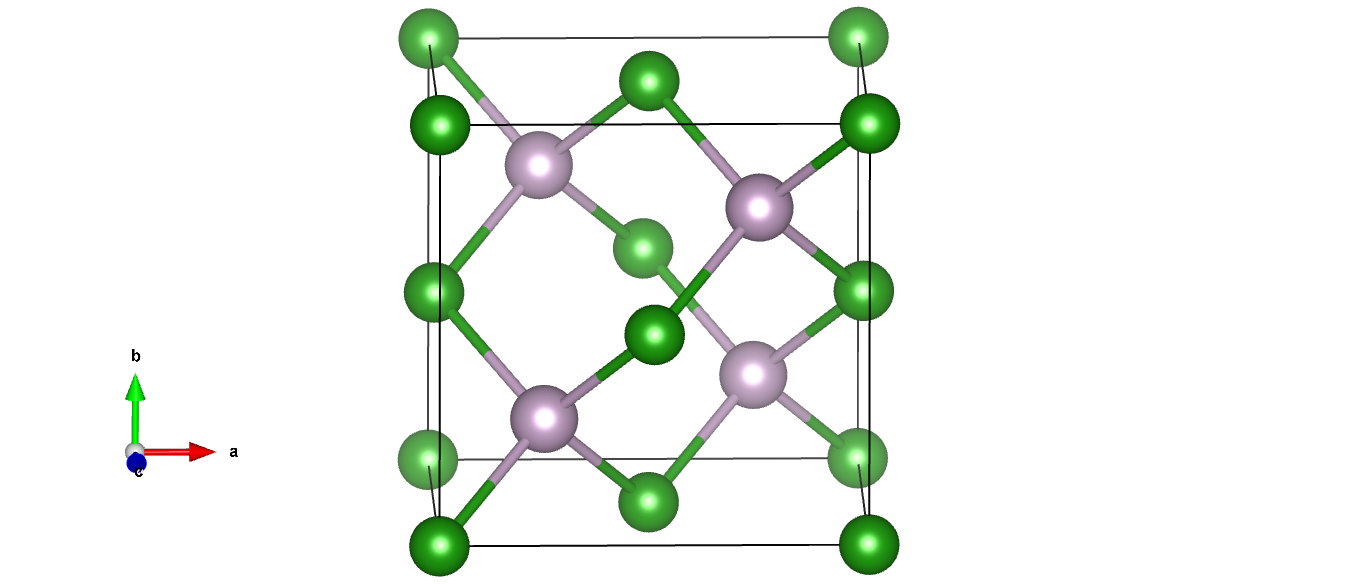


Structure of phase *F*-43*m*

*
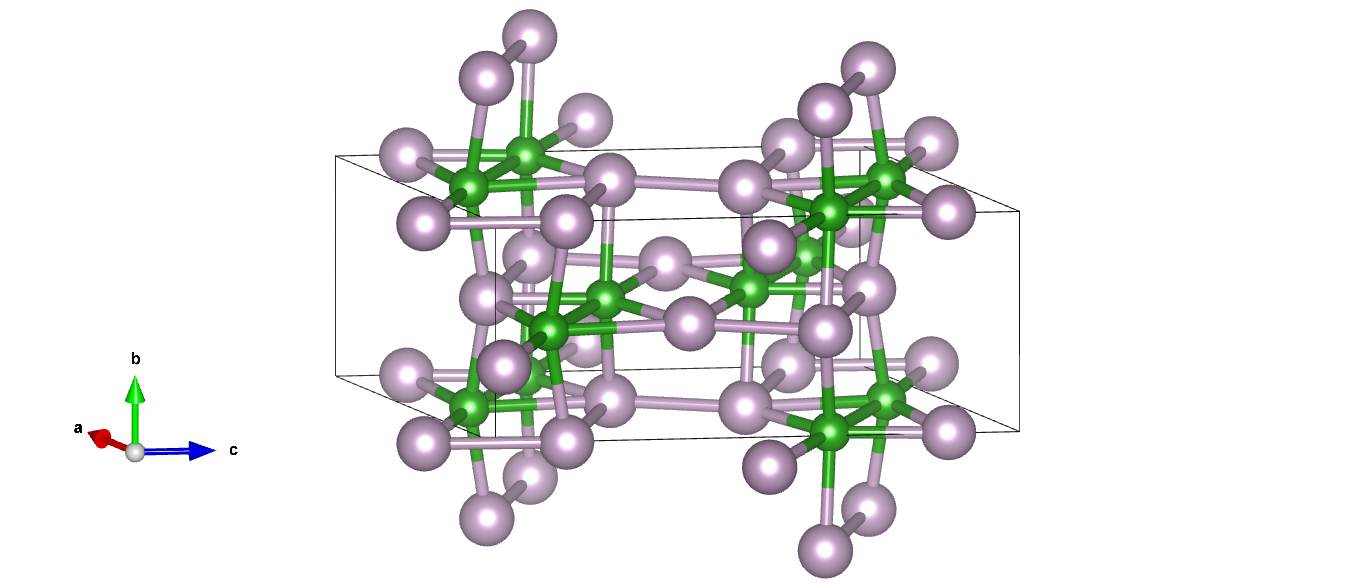
*

Structure of phase *C*2/*m* at 120 GPa

*
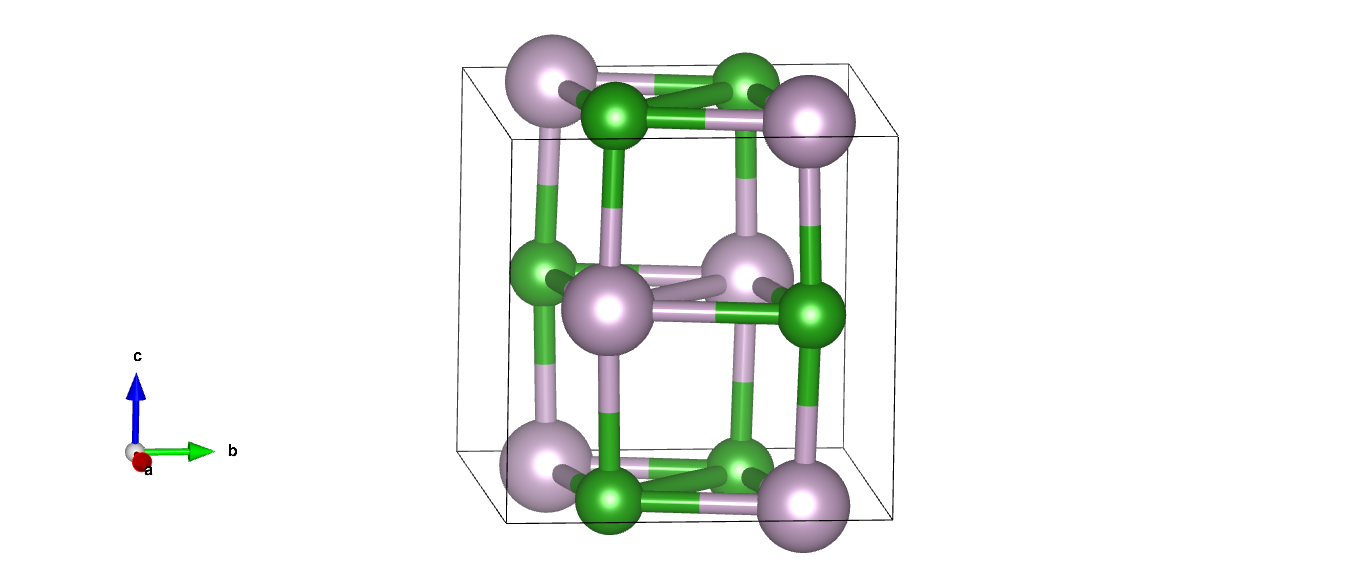
*

Structure of phase *P*4_2_/*mnm* at 210 GPa

*
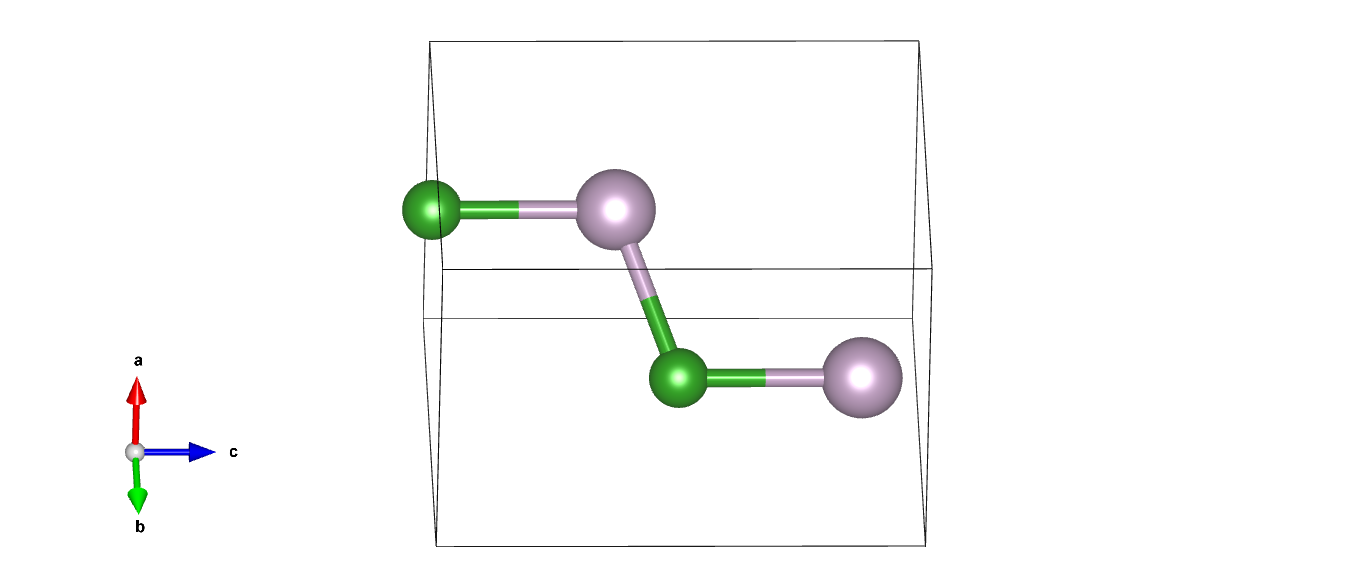
*

Structure of phase *P*6_3_*mc*

*
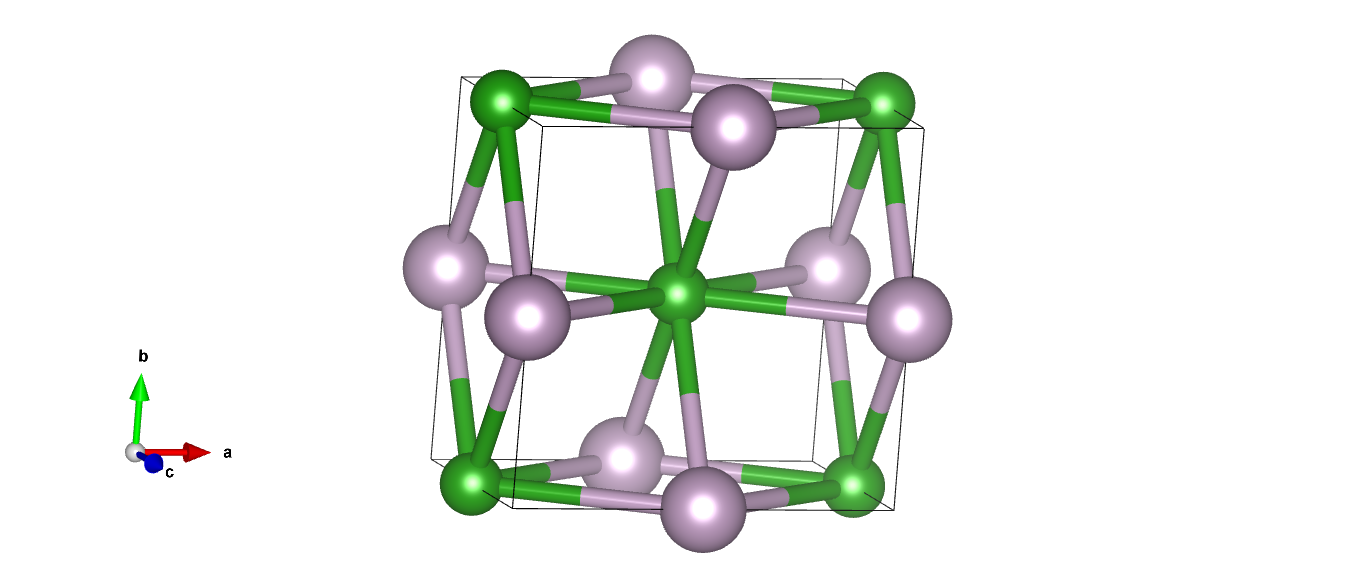
*

Structure of phase *Cmmm*

*
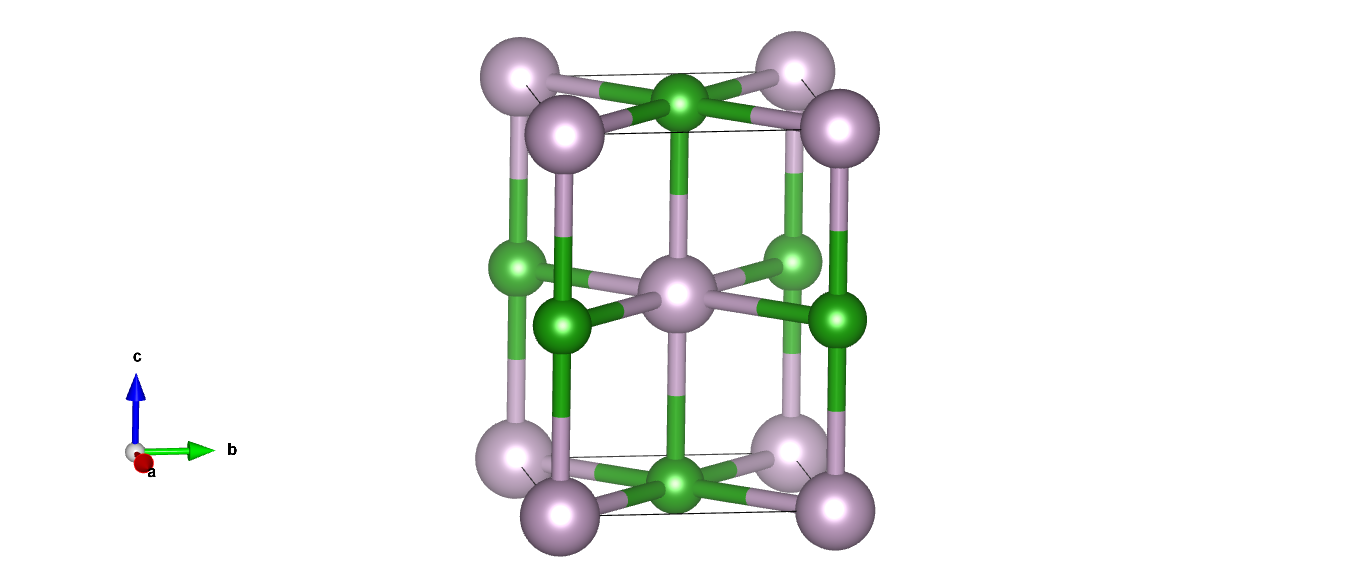
*

Structure of phase *Immm*

*
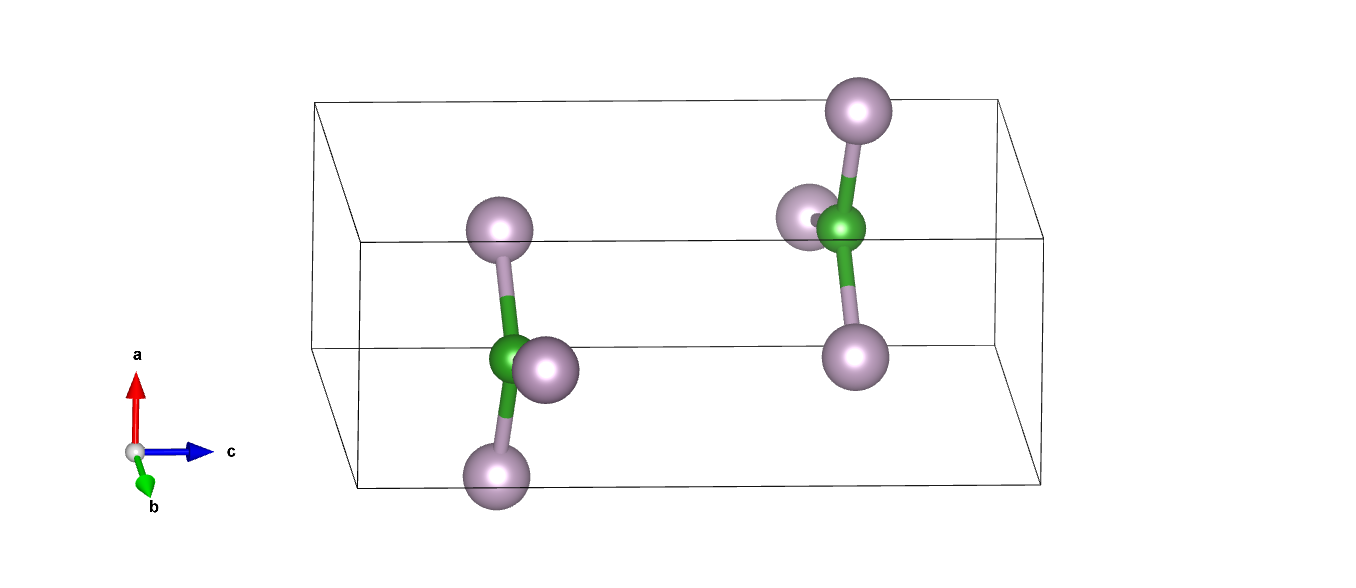
*

Structure of phase *P63/mmc-I*

*
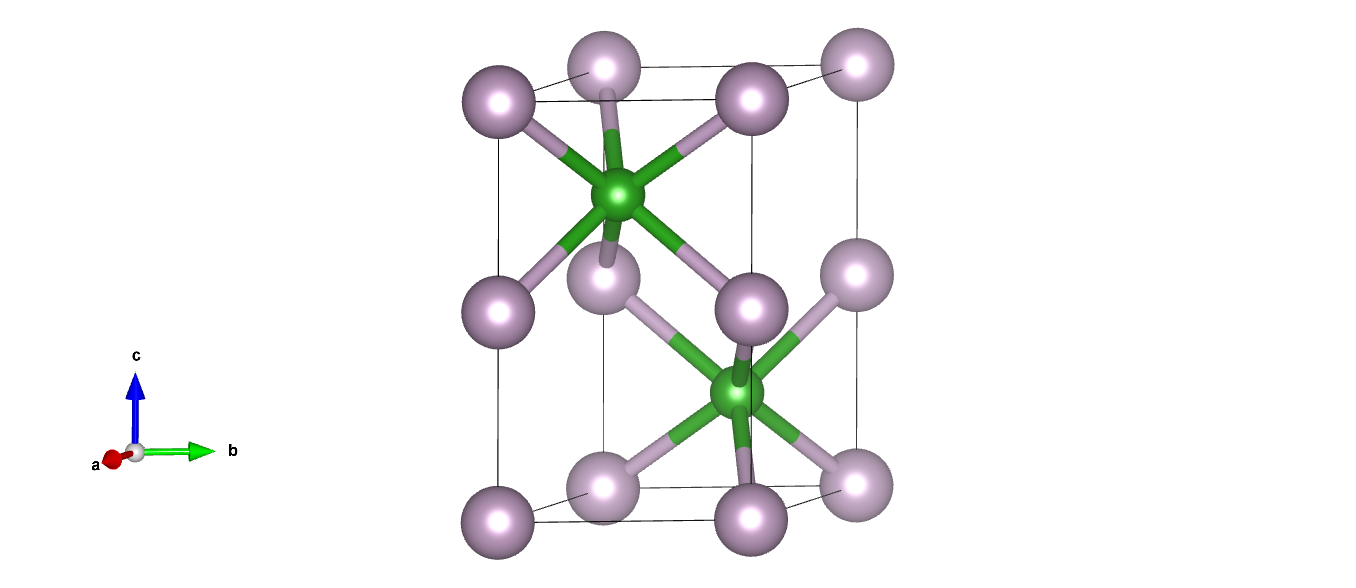
*

Structure of phase *P63/mmc-II*

*
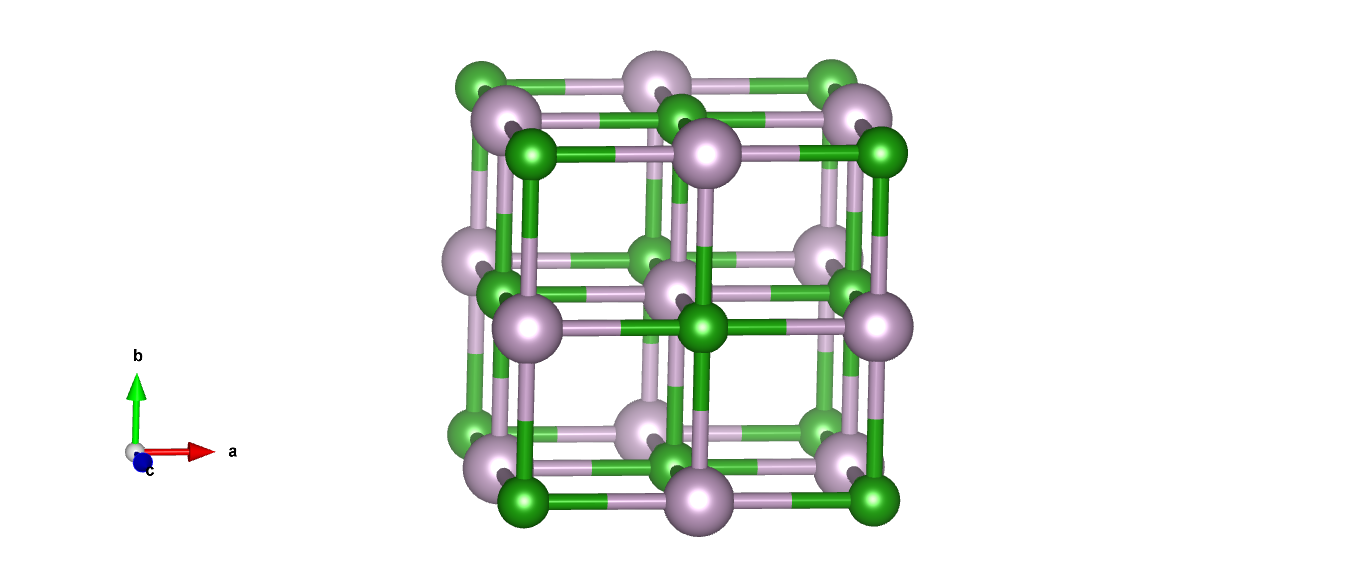
*

Structure of phase *Fm-3m*

**Figure S1.** The list of candidate structures with space groups *F*-43*m, C*2/*m, P*4_2_/*mnm, P*6_3_*mc, Cmmm, Immm, P63/mmc-I*, *P63/mmc-II*, and the reported high pressure BP rock salt structure *Fm-3m*.

**Table S1.** **Theoretical lattice constants, unit cell volume *V*, bulk modulus and first-order pressure derivative of bulk modulus (B´) at ambient pressure for *F*-43*m* structure. The other calculated and measured values are given for comparison.**

|  | Present work | | Other works | |
| --- | --- | --- | --- | --- |
|  | LDA | PBE | Theor. | Exp. |
| *a* (Å) | 4.492 | 4.547 | 4.501^a^,4.554^b^,  4.558^c^,4.51[^d^](#_ENREF_2),  4.546^e^ | 4.55 ^1^, 4.538 ^2^,  4.534 ^3^ |
| *V* (Å^3^) | 90.64 | 94.01 | 91.2 ^a^, 94.4^b^,  94.7^c^,91.7[^d^](#_ENREF_2),  93.9^e^ | 94.2^1^,93.45 ^2^,  93.21 ^3^ |
| *B* (GPa) | 175(Vinet),  173 (Murnaghan) ,  174 (BM) | 162(Vinet),  160 (Murnaghan) ,  161 (BM) | 176^a^,160^b^,  166[^c^](#_ENREF_1),172[^d^](#_ENREF_2),  170^e^ | 179 (Vinet) ^3^,  180 (Murnaghan) ^3^,  178 (BM) ^3^ |
| B´ | 3.76(Vinet),  3.67 (Murnaghan) ,  3.74 (BM) | 3.81(Vinet),  3.55 (Murnaghan) ,  3.75 (BM) | 3.68^a^,4.02^b^  3.7[^d^](#_ENREF_2),3.07^e^ | 3.3(Vinet) ^3^,  3.0 (Murnaghan) ^3^,  3.3 (BM) ^3^ |

a L/APW+lo-LDA Ref. ^4^

b L/APW+lo-GGA Ref. ^4^

c PWPP-LDA Ref. ^5^

d LMTO-LDA Ref. ^6^

e FP-LAPW-GGA Ref. ^7^

**References**

(1) Popper, P.; Ingles, T. A. *Nature* **1957**, *179*, 1075-1075.

(2) *Congres International de Chimie pure et applique, 16eme Paris 1957, Mem. Sect. (1958), 1957, 539-540* **1958**.

(3) Godec, Y. et al. *Journal of Superhard Materials* **2014**, *36*, 61-64.

(4) Meradji, H. et al. A. *Phys. Status. Solidi. B* **2004**, *241*, 2881-2885.

(5) Wentzcovitch, R. M.; Cohen, M. L.; Lam, P. K. *Phys. Rev. B* **1987**, *36*, 6058-6068.

(6) Lambrecht, W. R. L.; Segall, B. *Phys. Rev. B* **1991**, *43*, 7070-7085.

(7) Zaoui, A.; Hassan, F. E. H. *Journal of Physics: Condensed Matter* **2001**, *13*, 253.
